# Identification of risk profiles among persons attending a sexually transmitted infection clinic in Estonia

**DOI:** 10.1371/journal.pone.0353184

**Published:** 2026-07-13

**Authors:** Karolin Toompere, Krõõt Arbo, Ülo Maiväli, Made Laanpere, Maigi Eisen, Kirill Guskov, Anneli Uusküla

**Affiliations:** 1 Institute of Family Medicine and Public Health, University of Tartu, Tartu, Estonia; 2 Institute of Technology, University of Tartu, Tartu, Estonia; 3 Department of Obstetrics and Gynaecology, Institute of Clinical Medicine, University of Tartu, Tartu, Estonia; 4 Tartu University Hospital Women’s Clinic, Tartu, Estonia; 5 North Estonia Medical Centre, Tallinn, Estonia; Taipei Veterans General Hospital, TAIWAN

## Abstract

**Background:**

While generalized STI prevention guidelines exist, individuals present with diverse interconnecting risk behaviors and needs. This cross-sectional study used latent class analysis to identify distinct behavioural profiles among men and women undergoing STI testing and to examine their potential implications for STI prevention.

**Methods:**

Data was collected by questionnaire from 1171 individuals seeking STI testing at the North Estonia Medical Centre between August 2022 and October 2023. Latent class analysis was applied to identify subgroups based on eight relevant STI risk variables. Separate models were fitted to men and women.

**Results:**

We propose three latent classes for both sexes. Among men, three classes were identified: “multiple partners; elevated behavioural risk” (62.4%); “predominantly monogamous; fewer behavioural risk indicators” (21.9%); and, “multiple partners; previous STI history; concentrated co-occurring risk” (15.7%). Women were categorised as follows: “new sexual partners; inconsistent condom use” (41.6%); “multiple partners; previous STI history; concentrated behavioural risk” (30.2%); and, “predominantly monogamous; possible partner-related exposure” (28.2%). The identified profiles demonstrated heterogeneity in sexual behaviour, condom use, prior STI history, and substance use across STI clinic attendees.

**Conclusions:**

The identified behavioural profiles highlight how co-occurring risk factors cluster within STI clinic populations and may help contextualise prevention discussions alongside individualized clinical assessment. These findings may support prevention discussions addressing partner communication, condom use, substance use, IPV awareness, and partner notification.

## Introduction

Sexually transmitted infections (STIs) remain a concern in EU/EEA countries [1]. Recent evidence highlights rising case notification for Chlamydia trachomatis, Neisseria gonorrhoeae, and syphilis, as opposed to decreasing trends in non-EU/EEA countries [2,3].

Research in Europe and North America demonstrates that individuals seeking STI services are not a uniform group but comprise identifiable subgroups with varying, interconnected risk profiles [4–8]. Beyond infection risk, these individuals often experience co-occurring behavioural and social vulnerabilities, substance use, and higher rates of intimate partner violence (IPV) [6–14].

Traditional STI risk assessment often focuses on individual behavioural indicators, such as partner number, condom use, or substance use. However, these factors frequently co-occur in non-random ways, and individuals with similar single risk indicators may differ substantially in their broader behavioural patterns and prevention needs. Rather than replacing conventional risk assessment, latent class analysis may help characterise recurring patterns of co-occurring behavioural risk factors within clinical populations and thereby complement existing prevention approaches.

Previously, latent class analysis has been used to characterize sexual risk and substance-use behaviours in general populations [15–17]. Few studies [8] have examined sex‑specific latent classes in STI‑clinic populations, and even fewer of them have connected these subgroups to practical prevention needs. However, STI clinic attendees represent a distinct population characterised by higher baseline STI exposure, diverse testing motivations, and overlapping behavioural vulnerabilities.

This study aims to identify distinct behavioural profiles among STI clinic attendees using latent class analysis based on patterns of sexual behaviour, STI history, and substance use, and to explore how these profiles may inform more individualised STI prevention approaches.

## Materials and Methods

### Study design

This cross-sectional study used sex-stratified latent class analysis to identify patterns of STI-related risk behaviours.

### Setting

The North Estonia Medical Centre (NEMC), the largest healthcare provider in Estonia, serves approximately 647,200 individuals in Tallinn and surrounding areas [18]. Before August 2022, NEMC STI testing necessitated an appointment with a dermatovenerologist. A streamlined process was then implemented allowing individuals to request referral for STI laboratory tests (urine and blood) upon registration. Routine testing includes a paper-based questionnaire developed de novo by the Estonian Society for Dermatovenerologists. As a clinical screening tool rather than a reflective psychometric scale, it captures formative behavioral indicators, including sociodemographics, STI history, sexual behavior, substance use, and sexual violence, to inform clinical assessment. The instrument’s content validity was established through expert clinical review and alignment with international STI screening guidelines. These clinical responses served as the primary data source for this study. Test results are available within two days; positive results trigger a referral to a dermatovenerologist or infectious disease specialist. All questionnaires are anonymized by removing patient identification labels prior to data storage.

### Participants

Data were collected from individuals seeking STI testing, with no exclusion criteria, at the NEMC between August 2022 and October 2023. Data on all individuals who completed the risk assessment questionnaire (in Estonian, English, or Russian) are included in the study. Laboratory testing procedures are described in detail in Appendix I in S1 File.

All individuals undergoing STI testing at the NEMC who completed the risk assessment questionnaire during the study period were included. For the latent class analysis, individuals reporting no sexual intercourse in the past six months or with incomplete data on indicator variables were excluded.

This study utilised existing routinely collected anonymised data from the NEMC records; therefore ethical committee approval was not sought. Under the Estonian Personal Data Protection Act §6 and the EU General Data Protection Regulation (GDPR, EU 2016/679), anonymised data are not classified as personal data and do not require ethics committee approval. Prior to commencing the study, the University of Tartu Human Research Ethics Committee was consulted and confirmed via email that, according to the applicable regulations, ethics committee approval was not required for the processing of anonymised data. Data use was conducted in accordance with NEMC procedures and regulations.

### Measures

To identify latent subgroups with different patterns of risk exposure, we selected 8 of 22 variables contained within the clinical screening questionnaire described in the *Setting*. The selection was theoretically grounded in the theory of planned behaviour, focusing on key behavioural domains, such as partner number, condom use, substance use, and prior STI history, that shape health-related intentions and behavioral control. These indicators were chosen to capture formative heterogeneity in risk behaviour rather than clinical outcomes. A detailed justification for each selected variable is provided in Appendix II in S1 File. We collapsed response categories of some variables included in the LCA because the initial response categories had small counts, making LC models unfeasible. As men and women might exhibit different patterns of sexual behaviour and outcomes, we stratified our analysis by sex. STI positivity and experience of IPV were examined as distal outcomes, allowing assessment of how identified latent classes differed in clinically relevant outcomes.

### Statistical analysis

Medians, quartiles, range, absolute and relative frequencies were used to describe participants, Kruskal-Wallis test, Chi-square test and Fisher’s exact test were used to compare age distributions and sexual behaviour in men and women. We calculated exact binomial 95% confidence intervals for proportions.

LCA was used to identify unobserved subgroups of individuals with similar patterns of risk behaviour based on the selected indicator variables. A detailed description of LCA procedures and the assessment of associations between LC membership and distal outcomes – testing positive for any STI or experiencing IPV – is provided in Appendix I in S1 File.

To label LCs, we identified the response category associated with the highest STI risk for each indicator variable. For example, for the variable “Number of sexual partners in the last 6 months,” we chose the category “five or more partners” as the highest risk category. Labels were then assigned using the estimated probabilities and clustering patterns of the selected behavioural indicators, with emphasis on the most distinguishing characteristics of each class.

Descriptive statistics, p-values, and 95% CIs for proportions were calculated with Stata 17 [19]. LC analysis and models with distal outcome were estimated with Latent GOLD 6.0 [20]. LCA was conducted and reported in accordance with the guidance provided by Schreiber (2017) [21].

### Linking latent class profiles to prevention needs

To explore potential clinical relevance, we used a deductive mapping approach based on the dominant behavioural characteristics of each latent class. Prevention considerations were linked to the behavioural patterns most strongly represented within each class (e.g., substance use, inconsistent condom use, prior STI history). These recommendations are intended as illustrative and non-exclusive examples of how recurrent combinations of risk factors might inform prevention discussions rather than prescriptive intervention algorithms. Individual behavioural risk factors remain clinically relevant regardless of latent class membership.

## Results

### Study participants

The questionnaire was completed by 1171 patients. Median age was 33 years (range 16–82) (Table 1). Most participants were men (77%), and 94.5% reported sexual activity in the past 6 months, with 44% reporting 0–1 partners, 46% reporting 2–4 partners, and 10% reporting five or more partners. Age group distribution and sexual behaviours were similar between men and women (Table 1). Condom use with new or casual partners (*n* = 916) was inconsistent, with 78.7% reporting not always using condoms in the last 6 months. HIV/AIDS had been previously diagnosed in six men and one woman.

**Table 1:** Sociodemographic and behavioural characteristics of individuals undergoing STI testing.

	Men n = 899		Women n = 272		Total n = 1171		p-value
**Age (years),**							
median (IQR)	33	(27-41)	33	(26-41)	33	(27-41)	0.231^a^
Min-max	16-76		16-82		16-82		
**Age group, n (%)**							
16-24	144	(16.0)	60	(22.1)	204	(17.4)	0.125^b^
25-34	349	(38.9)	95	(34.9)	444	(37.9)	
35-44	275	(30.6)	76	(27.9)	351	(30.0)	
45-82	130	(14.5)	41	(15.1)	171	(14.6)	
Missing	1		0		1		
**Past history of STI, n (%)**							
No	576	(64.1)	186	(68.4)	762	(65.1)	0.199^b^
Yes	322	(35.9)	86	(31.6)	408	(34.9)	
Missing	1		0		1		
**Purpose of the STI testing, n (%)**							
Symptomatic	255	(28.6)	69	(25.7)	324	(27.9)	0.191^b^
New sexual partner	257	(28.8)	93	(34.6)	350	(30.1)	
Other	380	(42.6)	107	(39.8)	487	(41.9)	
Missing	7		3		10		
**Sexually active in the last 6 months, n (%)**							
No	48	(5.3)	16	(5.9)	64	(5.5)	0.730^b^
Yes	851	(94.7)	256	(94.1)	1,107	(94.5)	
**Number of sexual partners in the last 6 months, n (%)**							
0-1	364	(41.7)	132	(49.8)	496	(43.6)	0.067^b^
2-4	416	(47.7)	108	(40.8)	524	(46.1)	
5+	92	(10.6)	25	(9.4)	117	(10.3)	
Missing	27		7		34		
**Sex of the partners in the last 6 months, n (%)**							
Opposite sex	631	(74.2)	119	(46.5)	750	(67.8)	<0.001^b^
Same sex	184	(21.6)	131	(51.2)	315	(28.5)	
Opposite and same sex	35	(4.1)	6	(2.3)	41	(3.7)	
Missing	1		0		1		
NA	48		16		64		
**Sexual practices in the last 6 months, n (%)**							
Involving anal sex	193	(22.9)	54	(21.2)	247	(22.5)	0.571^b^
Not involving anal sex	651	(77.1)	201	(78.8)	852	(77.5)	
Missing	7		1		8		
NA	48		16		64		
**Condom use with new or casual partner(s) in the last 6 months, n (%)**							
Always	155	(18.5)	40	(15.9)	195	(17.9)	0.319^b^
No casual or new partner	127	(15.1)	47	(18.7)	174	(16.0)	
Not always	557	(66.4)	164	(65.3)	721	(66.1)	
Missing	12		5		17		
NA	48		16		64		
**Time elapsed since they had sex without a condom with a new or casual partner in the last 6 months, n (%)**							
Less than 3 weeks	240	(44.0)	65	(41.4)	305	(43.4)	0.255^b^
More than 3 weeks	305	(56.0)	92	(58.6)	397	(58.6)	
Missing	12		7		19		
NA	342		108		450		
**Illicit drug use in the last 6 months, n (%)**							
No	787	(88.0)	236	(88.1)	1,023	(88.0)	0.990^b^
Yes	107	(12.0)	32	(11.9)	139	(12.0)	
Missing	5		4		9		
**Ever experiencing intimate partner violence, n (%)**							
No	835	(93.9)	246	(91.8)	1,081	(93.4)	0.280^b^
Cannot tell	26	(2.9)	8	(3.0)	34	(2.9)	
Yes	28	(3.1)	14	(5.2)	42	(3.6)	
Missing	10		4		14		
**Ever had sex with a person living with HIV/AIDS, n (%)**							
No	889	(99.6)	268	(99.6)	1,157	(99.6)	0.999^c^
Yes	4	(0.4)	1	(0.4)	5	(0.4)	
Missing	6		3		9		
**Ever had sex with a person injecting drugs, n (%)**							
No	887	(99.2)	265	(98.9)	1,152	(99.1)	0.705^c^
Yes	7	(0.8)	3	(1.1)	10	(0.9)	

NA – not applicable; ^a^ Kruskal-Wallis test; ^b^ Chi-square test; ^c^ Fisher’s exact test.

Table 1 also provides information on drug use, IPV, and sexual history with individuals living with HIV/AIDS or injecting drugs.

### Latent class analysis (LCA)

LCA was performed using data from 1039 individuals, comprising 798 (76.8%) men and 241 (23.8%) women, with no notable differences in sociodemographic and behavioural characteristics between the LCA group and the whole cohort (Appendix III in S1 File). Based on the eight selected manifest variables, we fitted models with one-to-five LCs for men and women.

Model selection based on multiple information criteria (AIC, BIC, SABIC, CAIC and AIC(3)) consistently supported a three-class solution for men (Appendix IV in S1 File). For women, AIC and BIC suggested two to four classes. However, the three-class model was supported by SABIC, CAIC, and AIC(3) and revealed a more interpretable solution, which led us to adopt it for women. Two and 4 class solutions were examined in a sensitivity analysis (Table 6.1 in S1 File), and the corresponding classification for alternative models is presented in Table 6.2 in S1 File. The final models were with satisfactory discrimination between classes, with a classification error of 11% for women and 13% for men (classification tables: Appendix V in S1 File).

**Fig 1:**
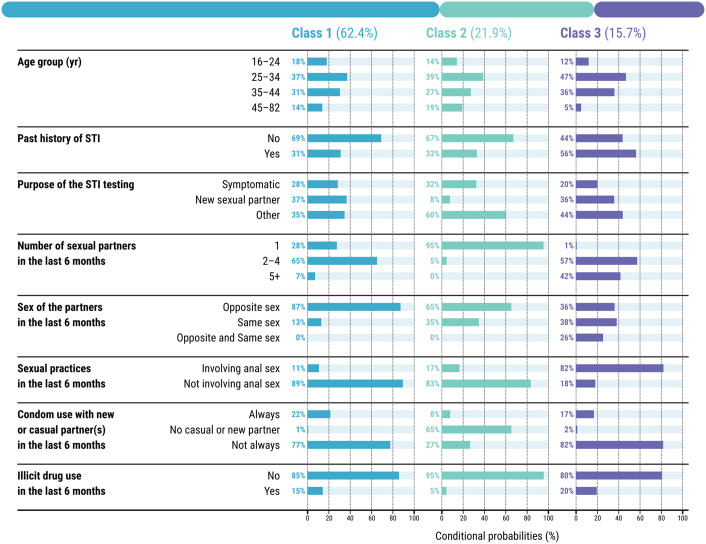
Latent Class conditional probabilities for men (*n* = 798).

The three classes identified for men are presented in Fig 1.

**Class 1** (“multiple partners; elevated behavioural risk”, *n* = 498, 62.4%) is the largest class and characterised by elevated rates of multiple sexual partnerships and inconsistent condom use, with moderate substance use and comparatively lower concentrations of previous STI history and overlapping sexual practices than Class 3.

**Class 2** (“predominantly monogamous; fewer behavioural risk indicators”, *n* = 175, 21.9%) is characterised by individuals reporting one sexual partner within the past six months (95.1%), with generally lower probabilities of behavioural risk indicators compared with the other male classes.

**Class 3** (“multiple partners; previous STI history; concentrated co-occurring risk”, *n* = 125, 15.7%) represented the subgroup with the greatest concentration of co-occurring high-risk characteristics, including previous STI diagnoses (56.2%), multiple and diverse partner types, anal sex practices, and the highest substance use prevalence among the three classes. They have multiple partners, engaging in diverse sexual practices with a mix of opposite-sex, same-sex, and both-sex partners (36.2%, 38.2%, and 25.6%, respectively). Notably, they exhibit the highest engagement in anal sex (81.8%) among the three classes, potentially contributing to their elevated STI risk. Substance use is also the highest in this group (19.6%).

**Fig 2:**
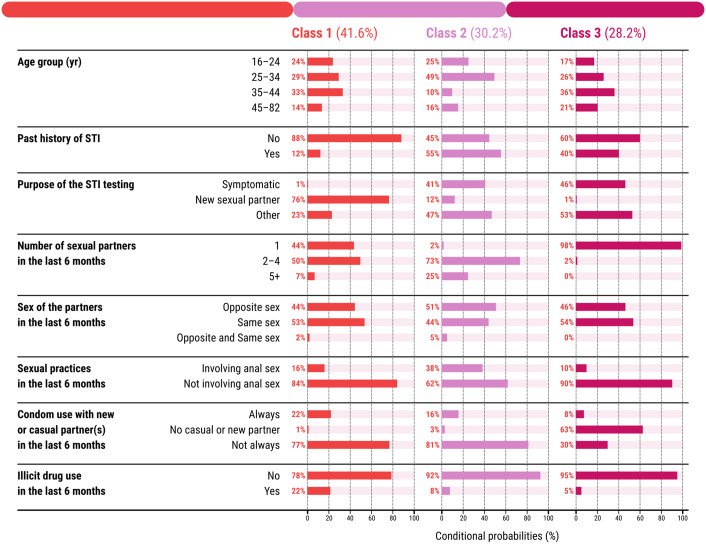
Latent Class conditional probabilities for women (*n*= 241).

For women, we identified three latent classes (Fig 2).

**Class 1** (“new sexual partners; inconsistent condom use”, *n* = 100, 41.6%). have no previous STI history (87.7%) and have either one (43.6%) or multiple partners (2–4, 49.5%) with a mix of opposite-sex (44.4%) and same-sex partners (53.4%) and lower engagement in anal sex (16.2%) compared to Class 2. Condom use is inconsistent, with 76.6% reporting not always using condoms with new or casual partners.

A new sexual partner (76.1%) is their likely reason for testing, suggesting greater STI risk awareness. This class has the highest prevalence of substance use among the three classes (21.6%).

**Class 2** (“multiple partners; previous STI history; concentrated behavioural risk”, *n* = 73, 30.2%) consists of mostly younger women (16–24 years, 25.1%; 25–34, 49.3%) with a high probability of previous STI diagnosis (55.4%), indicating frequent exposure to high-risk sexual behaviours. Testing is primarily driven by symptoms (40.8%) and other reasons (46.8%), besides new partners. These women have multiple partners (2–4, 73.2%; 5 + , 24.8%) with either opposite-sex (50.9%) or same-sex partners (43.9%), reflecting a more diverse sexual network, and a higher engagement in anal sex (38.3%) compared to other classes.

**Class 3** (“predominantly monogamous; possible partner-related exposure”, *n* = 68; 28.2%) are characterised by a high likelihood of monogamy (98.4% reporting one sexual partner in the last 6 months) and infrequent anal sex (10.1%), suggesting a preference for lower-risk sexual practices. However, the relatively high prevalence of previous STI diagnoses (40.2%) indicates potential past exposure to high-risk partners. The majority of these women (52.8%) sought STI testing for reasons beyond symptoms or new partners, indicating that partner notification or other unspecified factors might be influential in their decision to be tested. Whilst the majority have no new or casual partners (62.5%), most who did (79.5%) reported inconsistent condom use, highlighting a potential intervention area.

### Testing positive for STIs

Of the 1039 individuals in the analysis, 10.9% (95% CI 9.0–12.9%) tested positive for STIs (10.6%, 95% CI 8.6–13.0% of men and 11.6%, 95% CI 7.9–16.3% of women).

C. trachomatis infection was detected most often (5.3%, 95% CI 3.8–7.0% among men and 5.3%, 95% CI 2.9–9.0% of women), followed by M. genitalium infection (4.4%, 95% CI 3.1–6.0% among men and 2.9%, 95% CI 1.2–5.9% among women) (Appendix VII in S1 File).

Of the cohort, 0.5% (95% CI 0.2–1.1%) tested HIV-positive and 1.6% (95% CI 1.0–2.5%) were positive for HCV.

### STI proportions estimated from LCA

Estimated proportions of men testing positive for any STI for Class 1 (9.6%, 95% CI 7.0–13.0%) and Class 2 (9.7%, 95% CI 5.6–16.1%) are similar. Estimated proportion for Class 3 is higher (16.2%, 95% CI 10.0–25.2%) than Classes 1 and 2. Differences between all three Classes are not statistically significant (*p* = 0.20).

Among women, Class 2 (“multiple partners; previous STI history; concentrated behavioural risk”) had the highest estimated proportion of women testing positive for STIs (16.2%, 95% CI 9.0–27.4%), followed closely by Class 3 (13.2%, 95% CI 6.8–24.0%), while Class 1 (“new sexual partners; inconsistent condom use”) had a lower estimated prevalence (7.2%, 95% CI 3.3–15.1%). However, the differences between all the classes are not statistically significant (*p* = 0.27).

### Intimate partner violence estimated from LCA

The proportion of women who had ever experienced IPV was similar across Classes 1 (3.2%, 95% CI 0.9–11.1%) and 3 (2.9%, 95% CI 0.7–11.1%), and highest among Class 2 (9.4%, 95% CI 4.2–19.9%), respectively. However, the Class differences were not statistically significant (*p* = 0.58). Experience of IPV among men was 0.7% (95% CI 0.1–4.7%) in Class 1, 4.7% (95% CI 2.1–10.2%) in Class 2, and 11.9% (95% CI 6.9–19.8%) in Class 3.

### Prevention considerations across behavioural profiles (see Figs 3 and 4)

**Fig 3:**
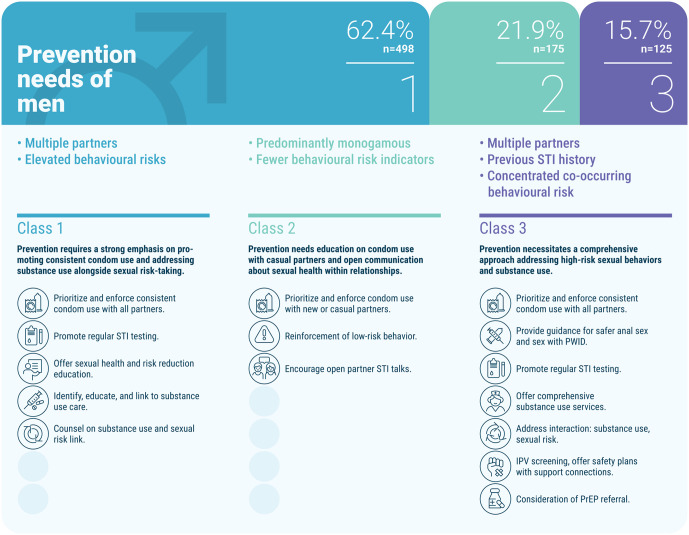
Prevention needs of men.

**Fig 4:**
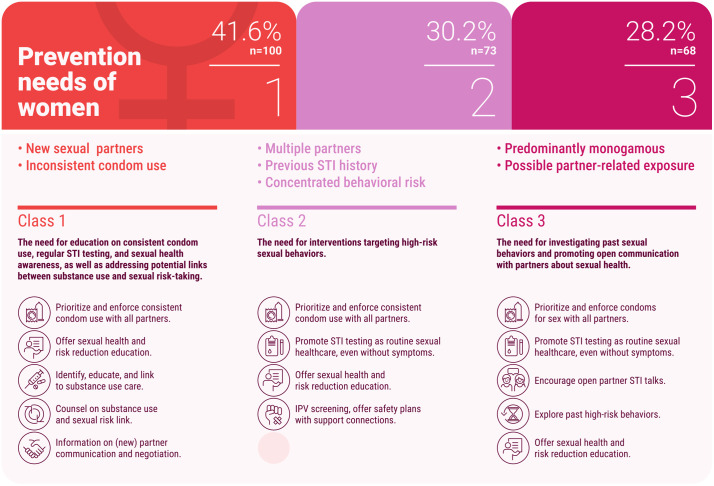
Prevention needs of women.

Prevention considerations frequently overlapped across classes because several behavioural risk factors were distributed across multiple groups. The prevention considerations presented in Figs 3 and 4 therefore reflect predominant behavioural patterns within classes rather than exclusive intervention pathways. The link between substance use and sexual risk-taking is acknowledged in both male and female Class 1, with recommendations for addressing substance use. Both men (Class 3) and women (Class 2) have recommendations for IPV screening, highlighting the importance of addressing violence and abuse in STI prevention for both sexes.

Key differences include women in Class 2 having a distinct focus on interventions targeting high-risk sexual behaviours; for women in Class 3, a specific emphasis is on investigating past sexual behaviours and promoting open communication with partners; men in Class 3 demonstrated overlapping behavioural risk indicators, including substance use and multiple sexual risk behaviours, suggesting the need for comprehensive prevention discussions; and men in Class 2 are characterised by a predominantly monogamous, low-risk profile, with prevention efforts focused on reinforcing these positive behaviours, but no direct parallel with women’s classes.

## Discussion

Our findings confirm significant heterogeneity among STI clinic attendees, identifying distinct subgroups with interconnecting risk profiles [6–8,13].

We identified a substantial proportion of individuals—15.7% of men and 30.2% of women—demonstrating a greater concentration of co-occurring behavioural vulnerabilities, including prior STI history and frequent substance use. These findings mirror high-risk patterns observed in Sweden (20% of men; 58% of women) [8] and, as expected, exceed proportions found in population-based studies in the US (10.4%) [16] and the Netherlands (7%) [17]. Our analysis adds a unique clinical dimension by incorporating variables specific to help-seeking populations, such as motivation for testing. Although some behavioural patterns identified in our study resemble those reported in STI clinic populations in other countries [6–8], the specific latent class structures observed here may be influenced by local healthcare systems, cultural factors, questionnaire design, and epidemiological context. Therefore, these similarities should not be interpreted as evidence that the same class structures are directly transferable to other settings. External validation in different populations is needed before broader applicability can be assumed.

### Behavioural risk profile variation across sex and age groups

Analysis of the three classes for men and women reveals common themes and gender differences in STI prevention needs. Condom use, regular testing, substance use, and open communication are important for both sexes. Among men, Classes 1 and 3 both reflected elevated behavioural risk patterns; however, Class 3 was distinguished by a greater concentration and co-occurrence of prior STI history, anal sex practices, diverse partner patterns, and substance use. Rather than representing entirely separate behavioural profiles, these classes appear to reflect differing intensities and clustering of overlapping risk characteristics, reinforcing that STI vulnerability exists along a continuum rather than within discrete “high-risk” and “low-risk” categories.

Among women, we identified profiles characterised by multiple co-occurring behavioural risk indicators, previous STI history, and partner-related vulnerability. Notably, one subgroup demonstrated relatively lower reported individual behavioural risk despite persistent STI positivity, suggesting that STI vulnerability may not be fully captured by individual behavioural indicators alone. Gender differences in testing motivations (new partners and symptoms among women versus more diverse reasons among men) further emphasise the need for context-sensitive prevention discussions.

STIs are increasingly diagnosed among older adults [22], highlighting the importance of recognising the heterogeneity within at-risk populations. Previous research demonstrates that middle-aged and older adults are a diverse group with varying needs [23], and our study supports this finding. Individuals from older age groups were represented across all latent classes, suggesting that age alone should not determine prevention approaches or assumptions regarding STI vulnerability.

### Intimate partner violence

The IPV patterns observed across classes did not fully align with the distribution of behavioural risk indicators. Among women, the highest IPV prevalence was observed in the class characterised by previous STI history and multiple partnerships rather than the predominantly monogamous class characterised by possible partner-related exposure. Among men, the class with broader elevated behavioural risk demonstrated lower IPV prevalence than the class with more concentrated overlapping vulnerabilities. Although these differences were not statistically significant, they highlight the complexity of interpersonal vulnerability within STI clinic populations and suggest that IPV risk may not correspond directly to behavioural STI risk patterns alone. These findings reinforce the importance of maintaining IPV awareness, screening practices, and appropriate referral pathways within STI services, particularly when overlapping behavioural and relational vulnerabilities are present [24,25].

### Interpretation and clinical implications of latent profiles

Although the latent classes demonstrated differing dominant behavioural patterns, overlap between classes was substantial, particularly among men. These findings suggest that the identified classes should not be interpreted as discrete clinical entities, but rather as probabilistic groupings reflecting predominant combinations and concentrations of co-occurring behaviours. Elevated behavioural risk characteristics were distributed across multiple classes, reinforcing that STI vulnerability exists along a continuum rather than within sharply separated “high-risk” and “low-risk” categories.

Accordingly, the latent classes are intended to complement rather than replace individualized assessment of specific risk factors during clinical care. We do not suggest that individual patients must be explicitly assigned to a class or that clinicians should use latent class algorithms in routine practice. The practical value of these findings is mainly at the population level and for STI service planning. Awareness of these recurring behavioural patterns may help clinicians recognise overlapping vulnerabilities that could otherwise be addressed in isolation, guide more focused history-taking and prevention counselling, reinforce partner notification practices, and inform appropriate referral pathways when relevant. The findings may also support the development of screening tools, staff training, and prevention activities.

Although some classes demonstrated higher point estimates for STI positivity and IPV, differences between classes were not statistically significant. The identified latent profiles should therefore be interpreted primarily as descriptive behavioural groupings rather than strongly discriminatory or outcome-predictive clinical categories. Nevertheless, the observed clustering of behavioural vulnerabilities may help support more integrated and context-sensitive STI prevention discussions within clinical services.

### Study strengths and limitations

The study included a substantial number of participants testing for STIs in a large hospital, encompassing a wide range of ages, lifestyles, and members of both sexes. The use of a structured risk assessment questionnaire allowed evaluation of multiple behavioural and clinical risk indicators within a real-world clinical population.

Several limitations should be considered. The latent class models were based primarily on routinely collected behavioural and clinical variables, and important psychosocial, interpersonal, and structural domains (including trauma history, relationship dynamics, and social determinants of health) were not measured and therefore could not be incorporated into the latent profiles. The use of an ‘other’ category for gender or sexual orientation may mask specific risks within minority subgroups. Recall- and self-report biases may affect accuracy as self-reported information was used, and as only complete cases were included in the LCA there is a possible risk of non-response bias. Our analysis also relies on the accurate selection of manifest variables to identify distinct groups. Finally, the study was conducted within a single healthcare system using a locally developed screening instrument; therefore, the identified class structures may not be directly generalizable to other settings and require external validation.

## Conclusion

Our findings demonstrate heterogeneity in patterns of sexual behaviour, prior STI history, and substance use among STI clinic attendees. The identified latent classes should be interpreted as descriptive behavioural profiles that help contextualise how STI-related risk factors cluster within clinical populations alongside standard individualized assessment.

Elevated behavioural risk characteristics were observed across multiple latent classes, although some groups demonstrated greater concentrations of overlapping vulnerabilities, including previous STI history, multiple partnerships, substance use, and inconsistent condom use. These findings support prevention approaches tailored to both individual risk factors and broader patterns of co-occurring behaviours.

## Supporting information

S1 File Supplementary. (DOCX)
