# Supplementary material for: Identification of risk profiles among persons attending a sexually transmitted infection clinic in Estonia
Source: PLoS One. 2026 Jul 13;21(7):e0353184. doi: 10.1371/journal.pone.0353184 (PMC13362120; doi:10.1371/journal.pone.0353184)
Supplement: S1 File — (DOCX) [file pone.0353184.s001.docx]

**S1. SUPPLEMENT**

Appendix I: Supplementary Methods (page 1)

Appendix II: Measured Variables Considered for Latent Class Analysis (page 2)

Appendix III. Sociodemographic and behavioral characteristics of individuals in the LCA analysis (page 3)

Appendix IV: Goodness-of-Fit Statistics for Latent Class Analyses with Different Numbers of Classes (page 4)

Appendix V: Classification Tables of Final Models for Men and Women (page 4)

Appendix VI: Sensitivity Analyses (page 5)

Appendix VII: Observed Test Positivity Rates for Syphilis, Gonorrhea, Chlamydia, Mycoplasma Genitalium, and Trichomoniasis among study participants, n (%) (page 6)

**Appendix I: Supplementary Methods.**

**Laboratory Testing Methods for Sexually Transmitted Infections (STIs)**

First-void urine samples were collected and tested for Neisseria gonorrhoeae, Chlamydia trachomatis, Mycoplasma genitalium, and Trichomonas vaginalis using Alinity m STI AMP Kit real-time PCR assay (Abbot, USA); syphilis reagin antibodies were detected in serum with NewBio Rapid Plasma Reagin test (Newmarket Biomedical Ltd, UK); syphilis IgM, IgG antibodies in serum with enzyme-linked immunosorbent assays (EUROIMMUN AG, Germany); and total antibodies with electrochemiluminescence immunoassay (ECLIA) (Roche Elecsys® Syphilis assay, Switzerland). The laboratory used an immunoblot test (Euroline, EUROIMMUNE, Germany) to confirm positive results. For HIV testing, the Xpert HIV-1 VL test (Cepheid, USA), and ECLIA for HCV testing (Roche Elecsys Anti-HCV II, Switzerland), were used.

**Latent Class Analyses (LCA) Procedures**

Models with one to five latent classes were estimated and compared using Akaike Information Criteria (AIC), Bayesian Information Criteria (BIC), third-order AIC (AIC(3)) – criterion recommended as more suitable than BIC and AIC in determining the number of latent classes [1], sample-size adjusted BIC (SABIC), which is suitable in case of moderate sample sizes [2], consistent AIC (CAIC), relative entropy, and clinical interpretability. Preference given to interpretable solutions with lower information criteria values.

Local independence was assessed using bivariate residuals (>4 indicating violation); where necessary, residual associations between indicators were permitted [3]. Models were estimated using maximum likelihood with multiple random starts to avoid local maxima.

We assessed the association between LC membership and distal outcomes: testing positive for any STI or IPV via two-step LC models [4]. The most widely used approach, the naive 3-step method or modal assignment, where people are assigned to the class with the largest posterior membership probability, has been shown to lead to biased results [5] because it does not incorporate the uncertainty of class allocation into the analysis. Therefore, we used the method recommended to relate LCs to categorical distal outcomes and covariates [6], where, in the first step, the LC model is estimated without external variables, and in the second step, the parameters of that measurement model are fixed, and an external covariate or distal outcome is added. Wald statistics and robust 95% CI were calculated for the estimated coefficients, and STI and IPV prevalences in LCs were predicted from these logistic models.

**Appendix II: Measured Variables Considered for Latent Class Analysis**

We selected the variables for LCA modelling by including factors that capture key dimensions of risk behaviour, such as age, past STI history, purpose of testing, number and sex of sexual partners, sexual practices, condom use, and illicit drug use. Our aim is to ensure content validity by including variables, that we think represent orthogonal dimensions of risk behaviour. The final variable set also aims for parsimony, balancing the inclusion of relevant factors with the need for a clear and interpretable model.

| **Original variable and response categories** | **Reasoning for variable selection or rejection for latent class membership** |
| --- | --- |
| **Age** (year) | Included as age groups, as a previously reported STI risk factor [7,8] |
| **Sex** (man; woman) | Not included, stratification variable |
| **Living with HIV** (yes; no) | Not included due to the small number of HIV+ individuals |
| **Past history of STI** (chlamydia; gonorrhoea; genital herpes; genital warts; syphilis; trichomoniasis; HIV/AIDS; other; urinary tract infection that is not caused by gonorrhea or chlamydia) | Included (yes, no), as previously reported STI risk factor [7] |
| **Purpose of the STI testing** (I have symptoms; no symptoms - I only want to be tested; I suspect a sexually transmitted disease or HIV infection; a new sexual partner; I was referred by another doctor or healthcare facility; someone recommended me to come; other) | Included (symptomatic, new sexual partner, other), as previously reported STI risk factor [7] |
| **Complaint** (discharge; sores/blisters; formations/warts on the genitals; itching; rash; bleeding; pain, problems urinating/painful urination; soreness during intercourse; other) | Not included, correlates with the purpose of current testing |
| **Sexually active in the last 6 months** (yes; no) | Not included, correlates with questions about sexual partners and practices in the last 6 months |
| **Number of sexual partners in the last 6 months** (1, 2, …10, more than 10) | Included (0-1, 2-4, 5+ partners), as a previously reported STI risk factor [7,8] |
| **Sex of the partners in the last 6 months** (men; women; both men and women) | Included (opposite sex, same-sex, opposite and same-sex), as a previously reported STI risk factor [9] |
| **Sexual practices used by the patient and their partner(s) during the last 6 months** (vaginal sex; oral sex; anal sex) | Included (practices involving anal sex, other), as a previously reported STI risk factor [8,10] |
| **Condom use with new or casual partner in the past 6 months** (always; mostly; sometimes; never; no new or casual partner) | Included (always, not always, no casual or new partner), as a previously reported STI risk factor [8,11] |
| **Time elapsed since they had sex without a condom with a new or casual partner** (less than 3 weeks; more than 3 weeks) | Not included, correlates with condom use with new or casual partner |
| **Antibiotics used in the last 6 months** | Not included, not an STI risk factor |
| **Allergies to medicines** | Not included, not an STI risk factor |
| **Ever experiencing intimate partner violence** (yes; no; cannot tell) | Added as an outcome of interest (distal variable) |
| **Ever had sex with a person injecting drugs** (yes; no) | Not included due to a small number of events |
| **Ever had sex with a person living with the HIV/AIDS** (yes; no) | Not included due to a small number of events |
| **Illicit drug use in the last 6 months** (yes; no) | Included (yes, no), as a previously reported STI risk factor [12] |
| **Ever used a needle to inject drugs** (yes; no) | Not included due to a small number of events |
| **Tattoos** (yes; no) | Not included, not an STI risk factor |
| **Vaccinated against hepatitis B** (yes; no, cannot tell) | Not included, not an STI risk factor |
| **Vaccinated with HPV vaccine** (yes; no; cannot tell) | Not included, not an STI risk factor |

**Appendix III. Sociodemographic and behavioral characteristics of individuals in the LCA analysis**

|  | **Men** | | **Women** | | **All** | |
| --- | --- | --- | --- | --- | --- | --- |
| **Age**  median (Q1–Q3)  min-max | 33  16–76 | (27–40) | 33  16–82 | (26–41) | 33  16–82 | (27–41) |
| **Age group, n (%)** |  |  |  |  |  |  |
| 16–24 | 131 | (16·4) | 54 | (22·4) | 185 | (17·8) |
| 25–34 | 312 | (39·1) | 83 | (34·4) | 395 | (38·0) |
| 35–44 | 245 | (30·7) | 65 | (27·0) | 310 | (29·8) |
| 45–82 | 110 | (13·8) | 39 | (16·2) | 149 | (14·3) |
| **Past history of STI, n (%)** |  |  |  |  |  |  |
| No | 515 | (64·5) | 161 | (66·8) | 676 | (65·1) |
| Yes | 283 | (35·5) | 80 | (33·2) | 363 | (34·9) |
| **Purpose of the STI testing, n (%)** |  |  |  |  |  |  |
| Symptomatic | 224 | (28·1) | 62 | (25·7) | 286 | (27·5) |
| New sexual partner | 242 | (30·3) | 86 | (35·7) | 328 | (31·6) |
| Other | 332 | (41·6) | 93 | (38·6) | 425 | (40·9) |
| **Sexually active in the last 6 months, n (%)** |  |  |  |  |  |  |
| Yes | 798 | (100·0) | 241 | (100·0) | 1,039 | (100·0) |
| **Number of sexual partners in the last 6 months, n (%)** |  |  |  |  |  |  |
| 1 | 305 | (38·2) | 112 | (46·5) | 417 | (40·1) |
| 2–4 | 404 | (50·6) | 104 | (43·2) | 508 | (48·9) |
| 5+ | 89 | (11·2) | 25 | (10·4) | 114 | (11·0) |
| **Sex of the partners in the last 6 months, n (%)** |  |  |  |  |  |  |
| Opposite sex | 592 | (74·2) | 113 | (46·9) | 705 | (67·9) |
| Same sex | 174 | (21·8) | 122 | (50·6) | 296 | (28·5) |
| Opposite and same-sex | 32 | (4·0) | 6 | (2·5) | 38 | (3·7) |
| **Sexual practices in the last 6 months, n (%)** |  |  |  |  |  |  |
| Involving anal sex | 186 | (23·3) | 51 | (21·2) | 237 | (22·8) |
| Not involving anal sex | 612 | (76·7) | 190 | (78·8) | 802 | (77·2) |
| **Condom use with new or casual partner(s) in the last 6 months, n (%)** |  |  |  |  |  |  |
| Always | 143 | (17·9) | 39 | (16·2) | 182 | (17·5) |
| No casual or new partner | 121 | (15·2) | 46 | (19·1) | 167 | (16·1) |
| Not always | 534 | (66·9) | 156 | (64·7) | 690 | (66·4) |
| **Time elapsed since had sex without a condom with a new or casual partner, n (%)** |  |  |  |  |  |  |
| Less than 3 weeks | 278 | (40·8) | 73 | (36·7) | 351 | (39·9) |
| More than 3 weeks | 403 | (59·2) | 126 | (63·3) | 529 | (60·1) |
| **Illicit drug use in the last 6 months, n (%)** |  |  |  |  |  |  |
| No | 693 | (86·8) | 210 | (87·1) | 903 | (86·9) |
| Yes | 105 | (13·2) | 31 | (12·9) | 136 | (13·1) |
| **Ever experiencing intimate partner violence, n (%)** |  |  |  |  |  |  |
| No | 746 | (93·8) | 220 | (91·7) | 966 | (93·3) |
| Cannot tell | 22 | (2·8) | 8 | (3·3) | 30 | (2·9) |
| Yes | 27 | (3·4) | 12 | (5·0) | 39 | (3·8) |
| **Ever had sex with a person living with the HIV/AIDS, n (%)** |  |  |  |  |  |  |
| No | 793 | (99·5) | 240 | (99·6) | 1,033 | (99·5) |
| Yes | 4 | (0·5) | 1 | (0·4) | 5 | (0·5) |
| **Ever had sex with a person injecting drugs, n (%)** |  |  |  |  |  |  |
| No | 792 | (99·2) | 237 | (98·8) | 1,029 | (99·1) |
| Yes | 6 | (0·8) | 3 | (1·3) | 9 | (0·9) |

**Appendix IV: Goodness-of-Fit Statistics for Latent Class Analyses with Different Numbers of Classes**

|  |  | **AIC** | **BIC** | **AIC(3)** | **SABIC** | **CAIC** | **Relative entropy** |
| --- | --- | --- | --- | --- | --- | --- | --- |
| **Men** | 1-class model | 10342.12 | 10431.08 | 10361.12 | 10370.74 | 10450.08 | 1 |
|  | 2-class model | 10081.53 | 10240.72 | 10115.53 | 10132.75 | 10274.72 | 0.790 |
|  | 3-class model | **9956.75** | **10186.17** | **10005.75** | **10030.57** | **10235.17** | 0.671 |
|  | 4-class model | 9958.78 | 10258.44 | 10022.78 | 10055.20 | 10322.44 | 0.618 |
|  | 5-class model | 9962.75 | 10332.63 | 10041.75 | 10081.76 | 10411.63 | 0.633 |
| **Women** | 1-class model | 3190.30 | 3266.96 | 3212.30 | 3197.23 | 3288.96 | 1 |
|  | 2-class model | 3116.68 | **3245.62** | 3153.68 | 3128.34 | 3282.62 | 0.764 |
|  | 3-class model | 3094.51 | 3275.72 | **3146.51** | **3110.89** | **3327.72** | 0.751 |
|  | 4-class model | **3091.03** | 3324.51 | 3158.16 | 3112.26 | 3391.64 | 0.753 |
|  | 5-class model | 3094.51 | 3380.26 | 3176.51 | 3120.34 | 3462.26 | 0.797 |

AIC - Akaike Information Criteria; BIC - Bayesian Information Criteria; AIC(3) – third order AIC; SABIC -sample size adjusted BIC; CAIC – consistent AIC

**Appendix V: Classification Tables of Final Models for Men and Women**

|  |  | **Class 1** | **Class 2** | **Class 3** | **Total** |
| --- | --- | --- | --- | --- | --- |
| **Men** |  |  |  |  |  |
|  | **Class 1** | 469.8 | 13.8 | 14.7 | 498.4 |
|  | **Class 2** | 42.8 | 131.6 | 0.3 | 174.7 |
|  | **Class 3** | 28.4 | 0.6 | 95.9 | 124.9 |
|  | **Total** | 541 | 146 | 111 | 798 |
| **Women** |  |  |  |  |  |
|  | **Class 1** | 86.6 | 8.1 | 5.7 | 100.4 |
|  | **Class 2** | 7.8 | 64.0 | 0.9 | 72.8 |
|  | **Class 3** | 3.6 | 0.9 | 63.4 | 67.8 |
|  | **Total** | 98 | 73 | 70 | 241 |

Rows represent the expected number of individuals in each latent class (sum of posterior probabilities); columns show the number of individuals assigned to each cluster based on their highest posterior probability (modal assignment).

**Appendix VI: Sensitivity Analyses**

**Table 6.1 Latent Class Conditional Probabilities (%) from 2-Class and 4-Class Models for Women**

|  | **2-class model** | | **4-class model** | | | | |  | |
| --- | --- | --- | --- | --- | --- | --- | --- | --- | --- |
| **Indicator** | **Class 1** | **Class 2** | **Class 1** | **Class 2** | **Class 3** | **Class 4** | **Overall** | |  |
| Class Size | 75.68 | 24.32 | 28.86 | 27.67 | 27.55 | 15.91 |  | |  |
| **Past History of STI** | | | | | | | | |  |
| No | 68.89 | 60.33 | 43.79 | 59.56 | 85.30 | 89.12 | 66.81 | |  |
| Yes | 31.11 | 39.67 | 56.21 | 40.44 | 14.70 | 10.88 | 33.19 | |  |
| **Age (years)** | | | | | | | | |  |
| 16-24 | 23.82 | 18.00 | 24.51 | 18.55 | 20.14 | 29.22 | 22.41 | |  |
| 25-34 | 37.39 | 25.24 | 51.13 | 24.96 | 35.76 | 18.36 | 34.44 | |  |
| 35-44 | 23.50 | 37.79 | 8.38 | 35.87 | 30.77 | 38.64 | 26.97 | |  |
| 45-82 | 15.29 | 18.97 | 15.97 | 20.63 | 13.33 | 13.78 | 16.18 | |  |
| **Illicit Drug Use in the last 6 months** | | | | | | | | |  |
| No | 85.35 | 92.69 | 93.44 | 93.85 | 99.87 | 41.98 | 87.14 | |  |
| Yes | 14.65 | 7.31 | 6.56 | 6.15 | 0.13 | 58.02 | 12.86 | |  |
| **Purpose of the STI Testing in the last 6 months** | | | | | | | | |  |
| New sexual partner | 46.26 | 2.76 | 11.54 | 0.43 | 71.62 | 78.57 | 35.68 | |  |
| Other | 33.56 | 54.25 | 48.91 | 54.77 | 21.95 | 20.55 | 38.59 | |  |
| Symptomatic | 20.18 | 42.99 | 39.55 | 44.80 | 6.43 | 0.89 | 25.73 | |  |
| **Sex of Sexual Partners** | | | | | | | | |  |
| Opposite & same sex | 3.28 | 0.02 | 5.58 | 0.01 | 0.01 | 5.49 | 2.49 | |  |
| Opposite sex | 47.88 | 43.81 | 51.27 | 47.22 | 58.69 | 17.93 | 46.89 | |  |
| Same sex | 48.84 | 56.17 | 43.15 | 52.77 | 41.30 | 76.58 | 50.62 | |  |
| **Number of sex partners in the last 6 months** | | | | | | | | |  |
| 1 | 30.68 | 95.64 | 1.59 | 98.42 | 55.01 | 22.77 | 46.47 | |  |
| 2-4 | 55.65 | 4.25 | 73.48 | 1.52 | 40.22 | 65.63 | 43.15 | |  |
| 5+ | 13.67 | 0.11 | 24.93 | 0.06 | 4.77 | 11.61 | 10.37 | |  |
| **Sexual Practices in the last 6 months** | | | | | | | | |  |
| Involving anal sex | 24.98 | 9.27 | 40.16 | 11.21 | 0.20 | 40.31 | 21.16 | |  |
| Not involving anal sex | 75.02 | 90.73 | 59.84 | 88.79 | 99.80 | 59.69 | 78.84 | |  |
| **Condom use with new or casual partner(s) in the last 6 months** | | | | | | | | |  |
| Always | 19.67 | 5.31 | 15.33 | 6.35 | 30.87 | 9.39 | 16.18 | |  |
| No casual or new partner | 1.09 | 75.10 | 3.31 | 63.51 | 0.46 | 2.69 | 19.09 | |  |
| Not always | 79.24 | 19.59 | 81.36 | 30.13 | 68.67 | 87.92 | 64.73 | |  |

**Table 6.2 Classification Tables of** **2-Class and 4-Class Models for Women**

|  |  | **Class 1** | **Class 2** | **Class 3** | **Class 4** | **Total** |
| --- | --- | --- | --- | --- | --- | --- |
| **2-class** |  |  |  |  |  |  |
|  | **Class 1** | 177.2 | 5.4 |  |  | **182.6** |
|  | **Class 2** | 8.8 | 49.6 |  |  | **58.4** |
|  | **Total** | 186 | 55 |  |  | **241** |
|  |  |  |  |  |  |  |
| **4-class** |  |  |  |  |  |  |
|  | **Class 1** | 62.1 | 0.8 | 3.4 | 3.3 | 69.6 |
|  | **Class 2** | 0.8 | 62.4 | 3.0 | 0.5 | 66.7 |
|  | **Class 3** | 5.6 | 5.6 | 54.0 | 1.2 | 66.4 |
|  | **Class 4** | 2.5 | 1.1 | 3.6 | 31.0 | 38.3 |
|  | **Total** | 71 | 70 | 64 | 36 | 241 |

Rows represent the expected number of individuals in each latent class (sum of posterior probabilities); columns show the number of individuals assigned to each cluster based on their highest posterior probability (modal assignment).

**Appendix VII: Observed Test Positivity Rates for Syphilis, Gonorrhea, Chlamydia, Mycoplasma Genitalium, and Trichomoniasis among study participants, n (%)**

|  | **Men (N=798)** | **Women (N=241)** | **All (N=1039)** |
| --- | --- | --- | --- |
| **Any STI** | 85 (10.7) | 28 (11.6) | 113 (10.9) |
| Syphilis | 8 (1.0) | 3 (1.2) | 11 (1.1) |
| Gonorrhea | 7 (0.9) | 5 (2.1) | 12 (1.2) |
| Chlamydia | 42 (5.3) | 13 (5.3) | 55 (5.3) |
| Mycoplasma genitalium | 35 (4.4) | 7 (2.9) | 42 (4.0) |
| Trichomoniasis | 3 (0.4) | 0 | 3 (0.3) |

Comment: The rates of STI in our study appear to be on the lower end of the range observed in some other STI clinic settings, particularly those serving a general population [13,14].

**References:**

1. Andrews RL, Currim IS. A Comparison of Segment Retention Criteria for Finite Mixture Logit Models. *J Mark Res* 2003;40(2):235–243. doi:10.1509/jmkr.40.2.235.19225
2. Yang CC. Evaluating latent class analysis models in qualitative phenotype identification. *Comput Stat Data Anal* 2006;50(4):1090-1104. doi:10.1016/j.csda.2004.11.004.
3. Vermunt JK, Magidson J. Technical Guide for Latent GOLD 5.1: Basic, Advanced, and Syntax 1. Belmont, MA: Statistical Innovations Inc 2016.
4. Bakk Z, Kuha J. Two-Step Estimation of Models Between Latent Classes and External Variables. *Psychometrika* 2018;83(4):871–892. doi:10.1007/s11336-017-9592-7
5. Bolck A, Croon M, Hagenaars J. Estimating latent structure models with categorical variables: One-step versus three-step estimators. *Polit Anal* 2004;12(1):3–27. doi:10.1093/PAN/MPH001
6. Bakk Z, Kuha J. Relating latent class membership to external variables: An overview. *Br J Math Stat Psychol* 2021;74(2):340–362. doi:10.1111/bmsp.12227
7. Götz HM, van Oeffelen LA, Hoebe CJPA, van Benthem BH. Regional differences in chlamydia and gonorrhoeae positivity rate among heterosexual STI clinic visitors in the Netherlands: Contribution of client and regional characteristics as assessed by cross-sectional surveillance data. *BMJ Open* 2019;9(1):e022793. doi:10.1136/bmjopen-2018-022793
8. Kounta CH, Chazelle E, Ousseine YM, Lot F, Velter A. Factors associated with bacterial sexually transmitted infection screening uptake and diagnosis among men who have sex with men in France. *BMC Infect Dis* 2024;24(1):1431. doi:10.1186/s12879-024-10310-6
9. 9. Glick SN, Morris M, Foxman B, *et al*. A Comparison of Sexual Behavior Patterns Among Men Who Have Sex With Men and Heterosexual Men and Women. *J Acquir Immune Defic Syndr* 2012;60(1):83–90. doi:10.1097/QAI.0b013e318247925e
10. Marcus U, Bremer V, Hamouda O, *et al*. Understanding Recent Increases in the Incidence of Sexually Transmitted Infections in Men Having Sex With Men: Changes in Risk Behavior From Risk Avoidance to Risk Reduction. *Sex Transm Dis* 2006; Jan;33(1):11–7. doi:10.1097/01.olq.0000187224.10428.31
11. Warner L, Stone KM, Macaluso M, Buehler JW, Austin HD. Condom Use and Risk of Gonorrhea and Chlamydia: A Systematic Review of Design and Measurement Factors Assessed in Epidemiologic Studies. *Sex Transm Dis* 2006; 33(1):36–51. doi:10.1097/01.olq.0000187908.42622.fd
12. Haider MR, Kingori C, Brown MJ, Battle-Fisher M, Chertok IA. Illicit drug use and sexually transmitted infections among young adults in the US: evidence from a nationally representative survey. *Int J STD AIDS* 2020;31(13):1238–46. doi:10.1177/0956462420950603
13. Gratrix J, Plitt S, Turnbull L, *et al*. Trichomonas vaginalis Prevalence and Correlates in Women and Men Attending STI Clinics in Western Canada. *Sex Transm Dis* 2017;44(10):627-629. doi:10.1097/OLQ.0000000000000650
14. Bigler D, Surial B, Hauser CV, *et al*. Prevalence of STIs and people’s satisfaction in a general population STI testing site in Bern, Switzerland. *Sex Transm Infect* 2023;99(4):268-271. doi:10.1136/sextrans-2022-055472
